# Depletion of Foxp3^+^ regulatory T cells is accompanied by an increase in the relative abundance of Firmicutes in the murine gut microbiome

**DOI:** 10.1111/imm.13158

**Published:** 2019-12-12

**Authors:** Jan Kehrmann, Laura Effenberg, Camilla Wilk, Davina Schoemer, Nhi Ngo Thi Phuong, Alexandra Adamczyk, Eva Pastille, René Scholtysik, Ludger Klein‐Hitpass, Robert Klopfleisch, Astrid M. Westendorf, Jan Buer

**Affiliations:** ^1^ Institute of Medical Microbiology University Hospital Essen University of Duisburg‐Essen Essen Germany; ^2^ Institute of Cell Biology (Cancer Research) University Hospital Essen University of Duisburg‐Essen Essen Germany; ^3^ Biochip Laboratory Institute for Cell Biology–Tumour Research University of Duisburg‐Essen Essen Germany; ^4^ Institute of Veterinary Pathology Freie Universität Berlin Berlin Germany

**Keywords:** Foxp3, gut, microbiome, microbiota, regulatory T cells

## Abstract

A reciprocal interaction exists between the gut microbiota and the immune system. Regulatory T (Treg) cells are important for controlling immune responses and for maintaining the intestinal homeostasis but their precise influence on the gut microbiota is unclear. We studied the effects of Treg cell depletion on inflammation of the intestinal mucosa and analysed the gut microbiota before and after depletion of Treg cells using the DEpletion of REGulatory T cells (DEREG) mouse model. DNA was extracted from stool samples of DEREG mice and wild‐type littermates at different time‐points before and after diphtheria toxin application to deplete Treg cells in DEREG mice. The V3/V4 region of the 16S rRNA gene was used for studying the gut microbiota with Illumina MiSeq paired ends sequencing. Multidimensional scaling separated the majority of gut microbiota samples from late time‐points after Treg cell depletion in DEREG mice from samples of early time‐points before Treg cell depletion in these mice and from gut microbiota samples of wild‐type mice. Treg cell depletion in DEREG mice was accompanied by an increase in the relative abundance of the phylum Firmicutes and by intestinal inflammation in DEREG mice 20 days after Treg cell depletion, indicating that Treg cells influence the gut microbiota composition. In addition, the variables cage, breeding and experiment number were associated with differences in the gut microbiota composition and these variables should be respected in murine studies.

## Graphical abstract

In this study we showed that temporal regulatory T (Treg) cell depletion in DEpletion of REGulatory T cells mice was accompanied by an increase of the relative abundance of the phylum Firmicutes of the gut microbiota, indicating that Treg cells affect the gut microbiota composition. In addition, Treg cell depletion was associated with intestinal inflammation. Furthermore, the variables cage, breeding and experiment number affected the gut microbiota composition and should be respected in murine gut microbiota studies

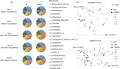

**Abbreviations**
ANCOM: analysis of composition of microbes
ASV: amplicon sequence variant
CD: cluster of differentiation
DEREG (mice): DEpletion of REGulatory T cells (mice)
DT: diphtheria toxin
Foxp3: Forkhead‐Box‐Protein P3
GFP: green fluorescent protein
ID: identifier
OTU: operational taxonomic unit
PC: principal coordinate
PCoA: principal coordinates analysis
permanova: permutational multivariate analysis of variance
QIIME: Quantitative Insights into Microbial Ecology
Reg III: regenerating islet‐derived protein 3
SEM: standard error of the mean
Treg cells: regulatory T cells
UniFrac (metric): unique fraction (metric)

## Introduction

The intestine contains a high abundance of forkhead box protein 3‐positive (Foxp3^+^) regulatory T (Treg) cells. They are fundamental for maintaining intestinal homeostasis by controlling immune responses of the innate and adaptive immune system.^1^ Loss‐of‐function mutations within *FOXP3*, the master transcription factor of Treg cells, occur in the IPEX (Immune dysregulation polyendocrinopathy enteropathy X‐linked) syndrome in humans, a fatal autoimmune disease with severe enteropathy, which is an early organ manifestation and a hallmark of the disease.^2^ The majority of Treg cells are derived from the thymus, but they can also be induced from naive CD4^+^ T cells by contact with antigens from commensal intestinal bacteria.^3^ Polysaccharide A derived from *Bacteroides fragilis* regulates CD4^+^ T‐cell differentiation towards Treg cells and enhances their activity.^4^, ^5^ Indigenous *Clostridium* species drive Treg cell accumulation by creating a transforming growth factor‐*β*‐rich environment. Furthermore, short‐chain fatty acids, fermentation products of commensal gut microbiota in the colon, in particular butyrate, were shown to regulate the Treg cell network by promoting the induction and fitness of Treg cells.^6^, ^7^, ^8^

Besides the impact of the gut microbiota to shape and regulate the maturation of the intestinal immune system, the immune system itself influences the gut microbiota composition. Treg cells control immune responses and can promote an immunoglobulin class switching to IgA.^9^ Secretion of antimicrobial peptides like defensins, RegIII and lysozymes directly affect the microbiota in the intestinal lumen.^10^, ^11^ However, studies analysing the impact of Treg cell depletion on the gut microbiota composition are lacking. We used the DEpletion of REGulatory T cells (DEREG) mouse model to study the effects of Treg cell depletion on intestinal inflammation and on altering the gut microbiota at different time‐points after depletion of Treg cells.

## Methods

#### Mice

The mice from the individual experiment with six DEREG mice and six wild‐type littermates were six male and six female mice from two breeding pairs at the age of 9–10 weeks at the start of the experiment. All animals used in this study were 7‐ to 10‐week‐old female and male DEREG mice and wild‐type littermates bred and housed under specific pathogen‐free conditions in the Laboratory Animal Facility of the University Hospital Essen. DEREG mice express the diphtheria toxin (DT) receptor and green fluorescent protein (GFP) under control of the *foxp3* promoter that allows for depletion of these cells after application of DT.^12^ All animal experiments were performed in accordance with institutional, state and federal guidelines (approved by the Landesamt für Natur, Umwelt und Verbraucherschutz North Rhine‐Westphalia, Germany; reference number: AZ 81‐02.04.2017.A456).

#### Protocols for depletion of Treg cells

##### Analysis of efficacy for depletion of Treg cells from the intestinal lamina propria

We injected DT intraperitoneally (30 ng/g body weight) twice weekly. Mice were killed at days 2, 9, 14 and 21. Lamina propria lymphocytes were isolated from the intestine of killed mice as described previously.^13^ Foxp3 expression was measured by detection of enhanced GFP. Cells were analysed by flow cytometry on an LSR II instrument using DIVA software (both from BD Biosciences, Franklin Lakes, NJ).

##### Study protocol for gut microbiota experiments

We injected DT intraperitoneally (30 ng/g body weight) at days 0, 4 and 7 in DEREG mice and non‐transgenic littermates. Stool samples were taken before Treg depletion (days −7 and 0), early after Treg depletion (day 5), late after Treg depletion (day 10) and after reconstitution of Treg cells (day 20). We did not apply DT for longer times because of the developing Treg cell rebound in the lamina propria of the intestine occuring despite repeated DT application. Retrobulbar blood samples were taken at days 0, 5, 7 and 20 to quantify the Foxp3^+^ Treg cell percentage from blood during the experiments.

#### Histology of the colon tissues

Histological examination of the colon was performed as described previously.^14^ Colons were rinsed with phosphate‐buffered saline, prepared as Swiss rolls and stored in 4% paraformaldehyde until the tissue was prepared for histological scoring. The colon tissues were assessed for immune cell infiltration of the lamina propria and tela submucosa and for hyperplasia and goblet cell loss.

#### DNA extraction and sequencing

DNA was extracted from a maximum of 200 mg stool per sample using the QIAamp DNA Stool Mini Kit (Qiagen, Hilden, Germany) according to the manufacturer's guidelines. Before DNA extraction, bead‐beating was performed with the stool samples resuspended in ASL buffer (Qiagen), using the FastPrep‐24 instrument (MP Biomedicals, Santa Ana, CA). Amplicon and index PCR were performed as described elsewhere.^15^ In brief, the V3/V4 region of the 16S rRNA gene was amplified using the 341F and the 785R primers of Klindworth *et al*.^16^ with an Illumina adapter overhang nucleotide sequence added 5′ of the locus‐specific sequences. PCR was performed with the Kapa HiFi HotStart ReadyMix (ThermoFisher Scientific, Waltham, MA), with 95° for 3 min and 25 cycles of 95° for 30 seconds, 60° for 30 seconds and 72° for 30 seconds with a final extension of 72° for 5 min. DNA from five negative preparation control samples (lysis buffer from the QIA DNA Stool Mini Kit undergoing the bead beating process and the entire DNA extraction procedure) and two PCR water samples were used as negative controls. PCR samples were run on a 1% agarose gel with a 100‐bp ladder to check for amplification efficacy. PCR products were cleaned up using the Qiagen PCR purification kit and eluted in 30 µl TE‐Buffer. Then, 2·5 µl of the purified PCR product was used as a template for the second round of PCR with the Kapa HiFi HotStart ReadyMix (ThermoFisher Scientific) using the N5XX and N7XX index primer of the Nextera XT Index Kit (Illumina, San Diego, CA). Each sample had a unique combination of N5XX and N7XX indices. PCR was performed with the following setting: 95° for 3 min and eight cycles of 95° for 30 seconds, 55° for 30 seconds and 72° for 30 seconds with a final extension of 72° for 5 min. PCR samples were run on a 1% agarose gel. For purification of the PCR products with the Qiagen PCR Purification Kit, seven and five individual pools were generated for separate Illumina Miseq runs containing similar amounts of PCR products as estimated by agarose gel electrophoresis. PCR products were eluted in 20 µl TE buffer. DNA concentration of the sample pools was measured using the Qubit High‐Sensitivity Assay (Life Technologies, Grand Island, NY, US). The sample pools were then combined to yield a single pool, which was quantified by quantitative PCR using the NEBNext Library Quant Assay (NEB, Ipswich, MA), and loaded on the flow cell at a concentration of 12 pm. A PhiX control library was spiked in at 3 pm concentration to increase sequence diversity, as recommended by Illumina. Sequencing was performed using the Illumina MiSeq 600 cycle reagent kit v3 (Illumina), with 301 cycles for read 1 and 2 and eight cycles for the two index reads.

#### Data analysis

Demultiplexed paired‐end fastq files and a mapping file were used as input files. Sequences were pre‐processed, quality filtered and analysed using the Quantitative Insights Into Microbial Ecology (QIIME) 2 pipeline (Version 2019.4).^17^ Adapters and primers were removed using cutadapt.^18^ We used the DADA2 software package^19^ wrapped in QIIME2 for modelling and correcting the Illumina fastq files including elimination of chimeras with the consensus method. Due to decreasing quality scores of bases at the end of the reads, we truncated 20 bases of the forward and 80 bases of the reverse reads. Data processing, quality filtering and removal of chimeras resulted in 3 906 621 sequences obtained from 60 samples of six DEREG and six wild‐type mice of one individual experiment and in 10 003 914 sequences obtained from all 25 DEREG mice and 11 wild‐type littermates from four individual experiments. The mean sequence frequency was 65 110, the maximum was 105 590 and the minimum number of reads was 30 626 for the experiment with six DEREG and six wild‐type mice. For the analyses of gut microbiota samples of mice from four experiments, the mean frequency was 54 133, the minimum frequency was 13 740 and the maximum frequency was 132 241.

Contaminating amplicon sequence variants (ASVs) were identified using the decontam R package with the ‘prevalence’ method using the default parameters.^20^ The identified ASVs were removed from all samples using the QIIME feature‐table filter‐features command. We used the q2‐diversity plugin for computing different *α* diversity metrics including Shannon's diversity index, Observed operational taxonomic units (OTUs) and Pielou's Evenness and *β* diversity metrics using weighted and unweighted UniFrac distance matrices with a sampling depth of 30 626 (one individual experiment with six DEREG and six wild‐type mice) and 13 740 (four independent experiments), which represent the lowest sequence number of the samples included in the analyses. Taxonomy was assigned using a pre‐trained Naïve Bayes classifier, which was trained on the greengenes 13_8_99% OTUs 16S rRNA gene full‐length sequences and the q2‐feature‐classifier plugin. Differential abundance testing was performed using ANCOM^21^ with the q2‐composition plugin. Associations between the categorical metadata columns and *α* diversity data were studied using the QIIME diversity *alpha‐group‐significance* command. We used the *qiime diversity beta‐group significance* command to perform permutational multivariate analysis of variance (permanova) of unweighted and weighted UniFrac distance matrices with 999 permutations to calculate *P*‐values and test for significant differences in *β* diversity among the groups. Kruskal–Wallis test with Dunn's multiple comparisons was used to estimate differences between groups for individual phyla and genera in graphpad prism, version 5.02 (GraphPad Software Inc., San Diego, CA).

## Results

### DT application transiently depletes intestinal Treg cells in DEREG mice and is associated with intestinal inflammation

**Figure 1:**
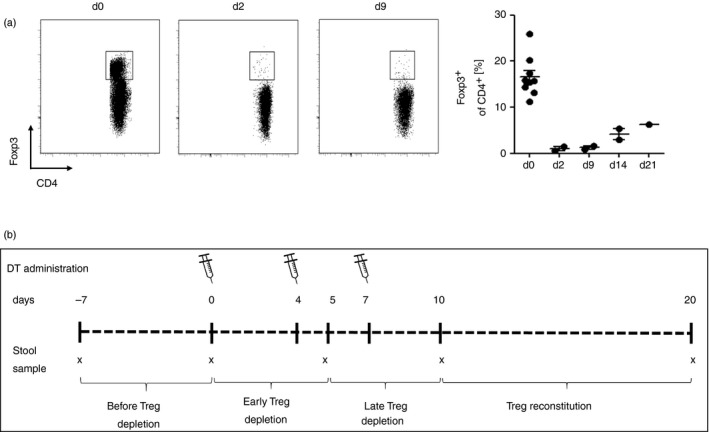
Administration of diphtheria toxin (DT) transiently depletes regulatory T (Treg) cells in the intestine of DEREG mice. (a) Efficacy of Treg cell depletion in colonic lamina propria. We administered DT twice weekly by intraperitoneal injection. Representative flow cytometric analysis illustrates percentage of Foxp3^+^ cells of CD4^+^ T cells. (b) Schematic time schedule of stool sampling and administration of DT for depletion of Foxp3^+^ Treg cells in gut microbiome analyses. Diphtheria toxin was administered intraperitoneally at days 0, 4 and 7 for depletion of Treg cells. Stool samples were taken before depletion of Treg cells (days −7 and 0), early after depletion of Treg cells (day 5), late after depletion of Treg cells (day 10) and after Treg cell reconstitution (day 20).

**Figure 2:**
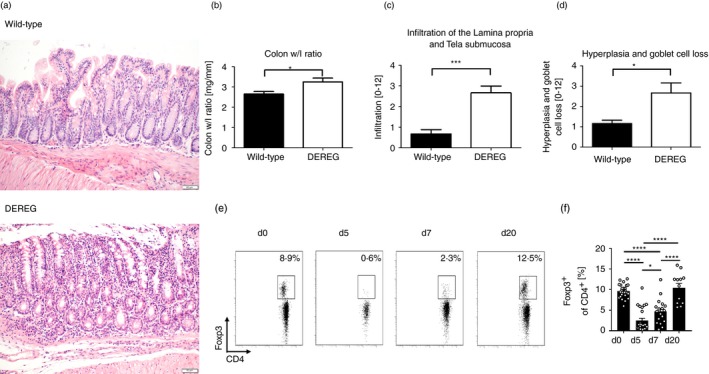
Histopathology of regulatory T (Treg) cell depleted DEREG mice and wild‐type littermates. (a) Representative tissue sections of the colon from Treg‐cell‐depleted DEREG mice and wild‐type littermates at day 20 were stained with haematoxylin & eosin (H&E) to assess pathology. Images show magnification at ×200. (b) Colon from Treg‐cell‐depleted mice and wild‐type littermates was prepared and colon weight/length ratio was measured. Bars represent the mean ± SEM. (c) Immune cells that infiltrate the lamina propria and tela submucosa were quantified and scored from H&E‐stained colonic tissue in DEREG mice and wild‐type littermates. Bars represent the mean ± SEM. (d) Goblet cells in periodic acid Schiff‐stained sections were counted and referred to villus length. Bars represent the mean ± SEM. Statistical significance was calculated using unpaired *t*‐test. (e) Representative flow cytometry plot shows percentage of Foxp3^+^ from CD4^+^ T cells measured in the blood taken from retrobulbar plexus at day 0 before DT administration and 5, 7 and 20 days after first DT administration. (f) Bars illustrate the mean percentage of Foxp3^+^ from CD4^+^ T cells ± standard deviation. Circles indicate percentage of Foxp3^+^ from CD4^+^ T cells of individual measures. Unpaired *t*‐test was used to test for statistical significance, *P < 0·05, ***P < 0·001, ****P < 0·0001.

First, we studied the efficacy of DT injection for depletion of Treg cells in the intestinal lamina propria of DEREG mice. DT application reduced the percentage of colonic Treg cells of CD4^+^ T cells from 16·5% before depletion of Treg cells to <1% at days 2 and 9 after the first DT application (Fig. 1a). A Treg cell rebound occurred between days 14 and 21 despite repeated DT application twice weekly. Due to the resulting Treg cell rebound occurring in the lamina propria, we concluded that the period for efficient Treg cell depletion in the colon was limited to a period of about 10 days after the first DT administration. Thereon, we developed our time schedule for the gut microbiota experiments, which is schematically illustrated in Fig. 1(b). We studied gut microbiota samples before Treg cell depletion (days −7 and 0), early after (day 5), late after depletion of Treg cells (day 10) and after reconstitution of Treg cells at day 20. We assessed the colon weight to length ratio and histopathological differences in six Treg‐cell‐depleted DEREG mice and six wild‐type littermates at day 20 of the experiment to analyse the graduation of intestinal inflammation. Interestingly, depletion of Treg cells was associated with low‐grade intestinal inflammation with significantly increased colon weight to length ratio, intestinal immune cell infiltration into the lamina propria and tela submucosa, hyperplasia and goblet cell loss in DEREG mice 20 days after DT application compared with DT‐treated wild‐type animals that did not exhibit comparable pathological histology (Fig. 2a–d). The efficacy of Treg cell depletion from blood was monitored during the experiment (Fig. 2e,f). No relevant signs of intestinal inflammation were detected early after depletion of Treg cells at day 7 (see Supplementary material, Fig. S1).

### Gut microbiota alterations in Treg‐cell‐depleted mice

**Figure 3:**
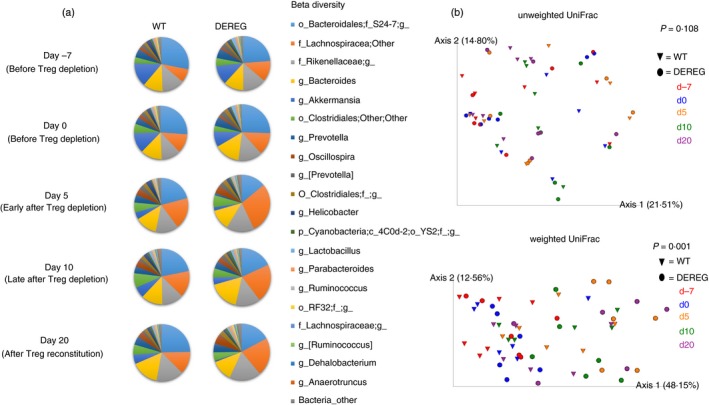
Gut microbiota composition in DEREG mice and wild‐type littermates at different time‐points before and after depletion of Treg cells. (a) The mean relative abundance of the 20 most abundant bacterial genera in DEREG mice and wild‐type littermates before Treg depletion at day −7 and 0, early after Treg depletion (day 5), late after Treg depletion (day 10) and after Treg reconstitution (day 20) is shown. f__;g__ represents genera that matched the Greengenes reference database, but could not be assigned below the order level; g__ represents operational taxonomic units (OTUs) that matched the Greengenes database and could not be assigned below the family level. (b) Principal coordinates analysis (PCoA) was performed using phylogenetic unweighted and weighted UniFrac distance matrices of samples of DEREG mice (circles) and wild‐type littermates (triangles). Samples of day −7 are illustrated in red, day 0 in blue, day 5 in orange, day 10 in green and day 20 in purple. Permutational multivariate analysis of variance (permanova) multivariate analysis was performed to test if samples within one group are more similar to each other then they are to samples from the other groups.

**Figure 4:**
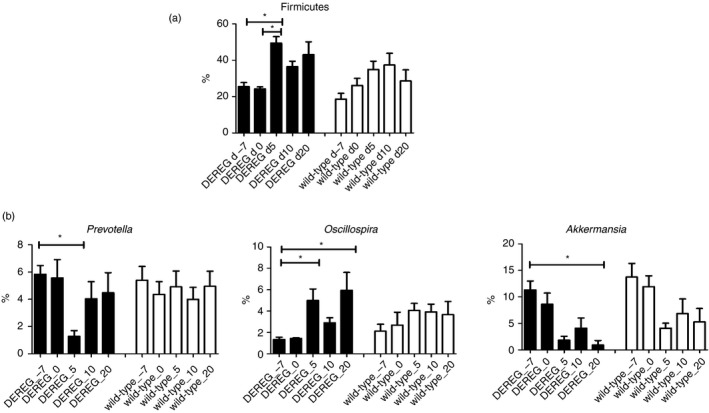
Relative frequency of different abundant phyla and genera. (a) Phyla and (b) genera with a relative frequency ≥1% that were differentially abundant between time‐points before and after Treg cell depletion in DEREG mice as identified by ANCOM. DEREG mice are illustrated by black bars and wild‐type mice by white bars. Kruskal–Wallis test with Dunn's multiple comparisons was used to calculate significance between different groups. Whiskers illustrate SEM.

We studied the effect of Treg cell depletion on the murine gut microbiota composition in an individual experiment with six DEREG mice and six wild‐type littermates. A genus of the *S24‐7* family of *Bacteroidales* and a genus of *Lachnospiraceae* were the two genera with the highest mean abundance in DEREG mice and wild‐type littermates (Fig. 3a). Principal coordinates analysis (PCoA) of unweighted and weighted UniFrac distance matrices did not clearly separate gut microbiota samples according to the mouse strain (DEREG or wild‐type) but Principal coordinate 1 (PC1) in PCoA of weighted UniFrac separated the majority of the DEREG gut microbiota samples of the time‐points after depletion of Treg cells from most of the DEREG samples of time‐points before depletion of Treg cells and of gut microbiota samples of wild‐type mice (Fig. 3b). We also performed PCoA for DEREG and wild‐type gut microbiome samples separately. Most DEREG samples of late time‐points after Treg cell depletion were separated from the samples of early time‐points before Treg cell depletion along PC1 that explained more than 50% of the variation between the samples (*P* < 0·001). PCoA of samples of the different time‐points in wild‐type littermates did not separate samples of late time‐points from early time‐points (*P* > 0·05; see Supplementary material, Fig. S2), suggesting that Treg cell depletion affects the gut microbiota composition. In contrast to unweighted UniFrac, which considers the absence and presence of organisms and is sensitive to differences in low‐abundance features, weighted UniFrac takes into account their abundance and is useful for examining differences in the community structure. At phylum level, the relative frequency of Firmicutes was significantly increased 5 days after Treg cell depletion in DEREG mice compared with time‐points before Treg cell depletion, while differences in wild‐type animals were not significant (Fig. 4a). A total of three genera with a relative abundance of at least 1% of the gut microbiota (*Prevotella*, *Akkermansia* and *Oscillospira*) were differently abundant between one time‐point before Treg cell depletion and at least one of the time‐points after Treg cell depletion (Fig. 4b). The alterations of the gut microbiome over time were not significant in wild‐type mice for these and other genera.

We also studied intra‐sample diversity metrics between individual mice and associated with Treg cell depletion. We compared the differences in Shannon diversity of the different time‐points before and after depletion of Treg cells within individual mice with the differences in Shannon diversity between mice. Shannon diversity did not vary significantly between individual mice for this individual experiment (see Supplementary material, Fig. S3A). The differences in Shannon diversity, observed OTUs and evenness between the different time‐points before and after depletion of Treg cells in DEREG mice were not significantly different when compared with the respective time‐points in wild‐type mice (see Supplementary material, Fig. S3B–D).

### Gut microbiota differences associated with cage, breeding, sex and experiment number

**Figure 5:**
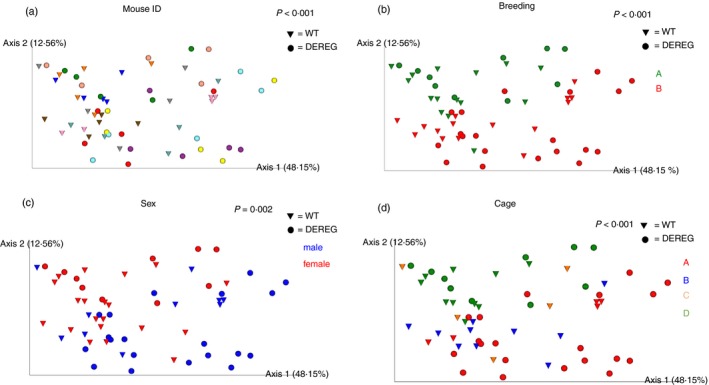
Principal coordinates analysis clusters gut microbiome samples according to the variables individual mouse identifier (ID), breeding, sex and cage. Principal coordinates analysis was performed using phylogenetic weighted UniFrac distance matrix. DEREG mice are illustrated as circles and wild‐type littermates as triangles throughout all parts of the figure. Permutational multivariate analysis of variance (permanova) of weighted UniFrac distance matrix with 999 permutations was used to analyse the strength and statistical significance for each category. (a) Each mouse ID is illustrated by an individual colour. (b) Mice from breeding A at the beginning of the experiment are illustrated in green and mice from breeding B in red. (c) Female mice are illustrated in red and male mice in blue. (d) Cage A is illustrated in red, cage B in blue, cage C in orange and cage D in green.

**Figure 6:**
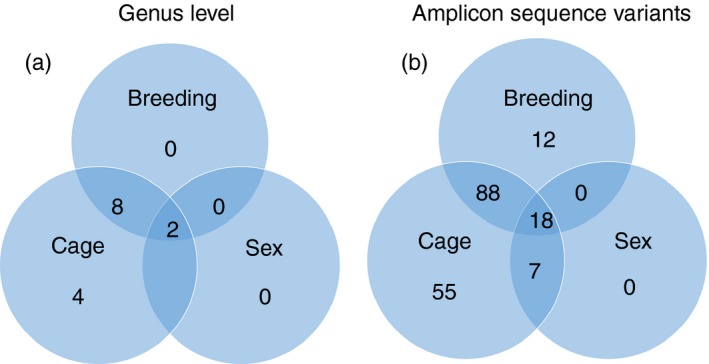
Differentially abundant amplicon sequence variants (ASVs) and genera linked to breeding, sex and cage number. Venn diagrams show the numbers of significantly different genera (a) and ASV (b) linked to and shared by the variables sex, breeding and cage.

The inter‐subject *β* diversity of the gut microbiota was significantly different as determined by PCoA using unweighted and weighted UniFrac distances (*P* < 0·001, Fig. 5a). The gut microbiota samples from different time‐points of individual mice clustered together in PCoA indicating that individual‐specific features were present in the gut microbiome. In addition, the factors cage, breeding and sex also separated the gut microbiota samples (*P* < 0·001 for breeding and experiment number, *P* = 0·002 for sex) in PCoA of weighted UniFrac distance matrices (Fig. 5b–d). At the genus level, 14 genera significantly differed for the three categories; four of these were unique for the variable cage (Fig. 6a, and see Supplementary material, Table S1). From a total of 918 ASVs, 180 were significantly different for the three categories, 55 were unique for the variable cage and 12 for breeding pair, while 10 other ASVs that differed between the categories were not restricted to a single category (Fig. 6b, and see Supplementary material, Table S2). Sex was not associated with significant differences at genus level and ASV level that were exclusively present for this category.

To test if the observed differences in the gut microbiota composition in DEREG mice are robust between individual experiments, we performed three more experiments with an additional number of 19 DEREG mice and five wild‐type littermates. When analysing the stool samples of the 25 DEREG mice of the four experiments, the relative abundance of the phylum Firmicutes was also significantly higher 5 days after Treg cell depletion as was the case in the individual experiment with six DEREG and six wild‐type animals (see Supplementary material, Fig. S4). For wild‐type animals of all four experiments, the relative abundance of the phylum Firmicutes was not significantly altered between the different time‐points before and after Treg cell depletion.

Analyses of genus level differences between the different time‐points before and after Treg cell depletion in DEREG mice for all experiments were not significant (data not shown). The variation of the gut microbiota diversity between the experiments varied significantly and was higher than the variation of the gut microbiota within the individual experiment with six DEREG and six wild‐type mice (see Supplementary material, Figs S5, S6). Experiment number was the variable with the highest number of unique genera and ASVs. (see Supplementary material, Fig. S7).

## Discussion

We found that multidimensional scaling separated most gut microbiota samples of DEREG mice from time‐points after Treg cell depletion from those DEREG samples before Treg cell depletion and from wild‐type gut microbiota samples. Furthermore, temporal Treg cell depletion in DEREG mice was accompanied by an increase in the relative abundance of the phylum Firmicutes and by intestinal inflammation of the colon tissue 20 days after Treg cell depletion. In addition, the variables cage, breeding and experiment number were associated with alterations of the gut microbiome.

Treg cells are a major regulator of intestinal homeostasis. Their importance becomes obvious in scurfy mice that lack functional Treg cells and exhibit severe signs of intestinal inflammation.^22^ We found increased colonic inflammation after the depletion of Treg cells in the DEREG mouse model at day 20, which is in line with others.^23^ We did not observe intestinal inflammation in the histology of the colon tissue 7 days after the first DT application, indicating that colon inflammation is a long‐term effect starting at a later time‐point after Treg cell depletion after the relative increase of the relative abundance of the phylum Firmicutes.

It has been shown that a reciprocal interaction exists between the immune system and the gut microbiota. Antibodies from single plasma cell clones have been reported to bind a broad but defined subset of commensal bacteria.^24^ Mice lacking T cells or T and B cells were shown to exhibit reduced gut microbiota diversity compared with wild‐type animals^25^ and the adoptive transfer of Foxp3^+^ Treg cells reconstituted diversity in CD3e^−/−^ T‐cell‐lacking mice. Treg cells influence the gut microbiota composition by regulating the secretion of IgA, antimicrobial peptides and pro‐inflammatory as well as anti‐inflammatory cytokines,^9^, ^26^, ^27^ and are, conversely, induced by several bacterial species.^28^ We found a shift of the DEREG gut microbiome samples after Treg cell depletion along PC1 in PCoA of weighted UniFrac and a significant increase in the relative abundance of Firmicutes in DEREG mice 5 days after Treg cell depletion, supporting the hypothesis that Treg cells shape the gut microbiota composition. The relative frequency of three genera in DEREG mice with a relative abundance of at least 1% of the total reads differed before and after depletion of Treg cells in the DEREG mice of a single experiment with an equal number of DEREG and wild‐type mice. When analysing the microbiome data of DEREG mice from four individual experiments, the relative abundance of the phylum Firmicutes also significantly increased after Treg cell depletion in DEREG mice in contrast to DT‐treated wild‐type animals. Although there was no significant tendency for changes in the gut microbiome over time over the 4 weeks of the experiment, this does not explain the differences for the phylum Firmicutes and the separation in multidimensional scaling of weighted UniFrac that we found associated with Treg cell depletion.

For the three genera that were significantly different for the individual experiment with six DEREG animals and six wild‐type littermates, significant differences did not exist when analysing the 25 DEREG mice from the four individual experiments, so we cannot exclude that this effect has been an experiment‐specific feature.

In addition, we found significant inter‐individual variation in the murine gut microbiome, a finding that has been reported previously.^29^ Furthermore, the variables cage, breeding and experiment number were variables that were associated with significant differences in the gut microbiota composition, which is in line with other murine studies.^30^, ^31^, ^32^ According to the number of significantly different abundant genera, the variation of the gut microbiota between the cages and individual experiments had the highest impact on the gut microbiota in our study. Significant variation of the murine gut microbiota between animals of different cages has been reported previously.^33^, ^34^ Some differing ASVs and genera were only present for the variable breeding, indicating that the variable also has an impact on the gut microbiota composition. The DEREG mouse model is advantageous for studying the impact of Treg cells on the gut microbiota, as it allows for specific depletion of Treg cells^23^ and for tracking of alterations of the gut microbiota in individual mice over time before and after depletion of Treg cells. This allows for detection of effects caused by the depletion of Treg cells in spite of the existence of other variables that influence the gut microbiota composition and could superpose the effects of Treg cell depletion on the gut microbiome when choosing non‐longitudinal approaches.

The DEREG mouse model is limited by the temporal depletion of Treg cells, as a rebound of these cells occurs in the intestine despite repeated DT application. This Treg cell rebound limits the model to study effects of long‐term Treg cell depletion on the gut microbiota.

## Conclusions

Our data showed that Treg cell depletion in DEREG mice was accompanied by an increase in the relative abundance of the phylum Firmicutes of the gut microbiota and by subsequent colonic inflammation. In addition, the variables cage, breeding and experimental variation affected the gut microbiota composition and should be regarded as influencing the gut microbiota composition in murine studies.

## Disclosures

The authors declare that they have no conflict of interest.

## Supporting information


**Figure S1.** Histopathology of colon tissues 7 days after diphtheria toxin application

**Figure S2.** Principal coordinates analysis separates gut microbiota samples of late time points after diphtheria toxin application from time‐points before diphtheria toxin application in DEREG mice but not in wild‐type littermates.

**Figure S3.** Illustration of *α* diversity metrics over time in DEREG and wild‐type mice.

**Figure S4.** Analysis of the gut microbiota composition and differences in *β* diversity between 25 DEREG and 11 wild‐type mice of four individual experiments.

**Figure S5.** Illustration of *α* diversity metrics over time in DEREG and wild‐type mice of four individual experiments.

**Figure S6.** Principal coordinates analysis clusters gut microbiome samples according to individual mouse identifier (ID), age, sex and experiment number.

**Figure S7.** Differentially abundant amplicon sequence variants and genera linked to age, sex and experiment number.

**Table S1.** Differentially abundant genera according to the variables breeding, sex and cage.

**Table S2.** Differentially abundant amplicon sequence variants (ASV) according to the variables breeding, sex and cage.

**Table S3.** Differentially abundant genera according to the variables age, sex and experiment number in 25 DEREG mice and 11 wild‐type littermates.

**Table S4.** Differentially abundant amplicon sequence variants (ASV) according to the variables age, sex and experiment number in 25 DEREG mice and 11 wild‐type littermates.
