# Supplementary figures and images for: Depletion of Foxp3+ regulatory T cells is accompanied by an increase in the relative abundance of Firmicutes in the murine gut microbiome
Source: Immunology. 2019 Dec 12;159(3):344–53. doi: 10.1111/imm.13158 (PMC7011623; doi:10.1111/imm.13158)

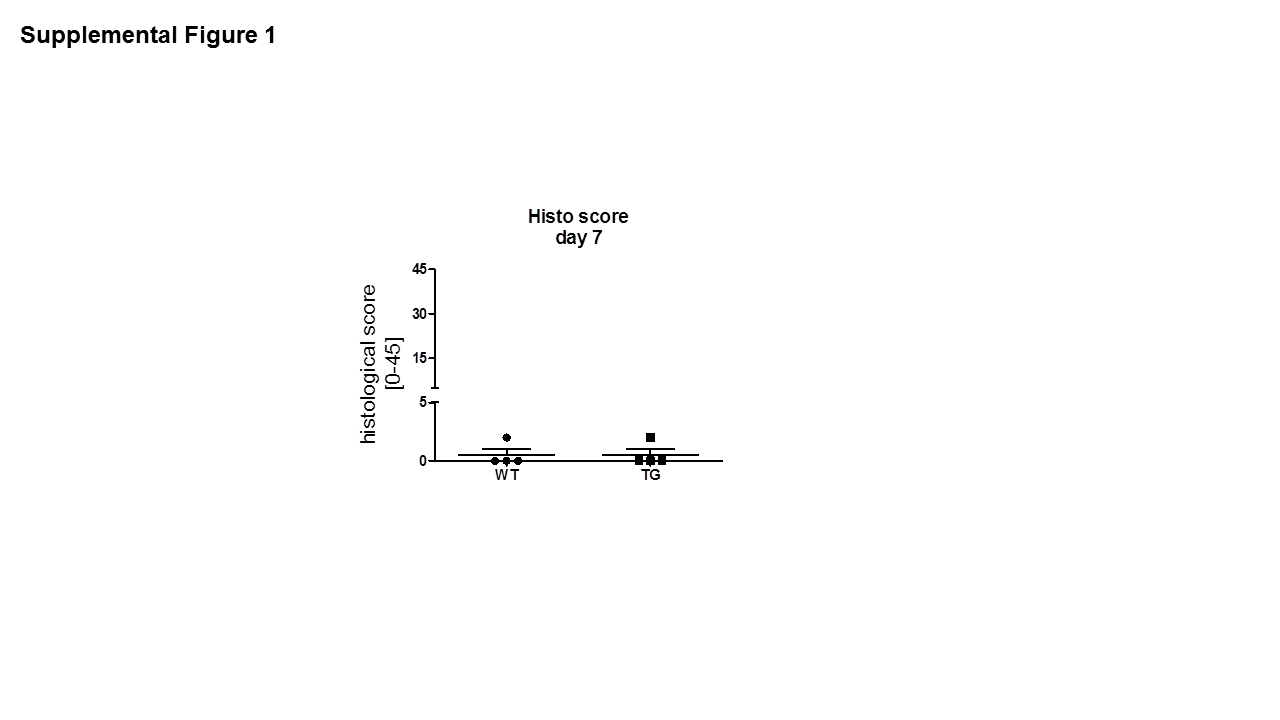

Supplement: Supplementary file 1 — Figure S1. Histopathology of colon tissues 7 days after diphtheria toxin application [file IMM-159-344-s001.TIF]

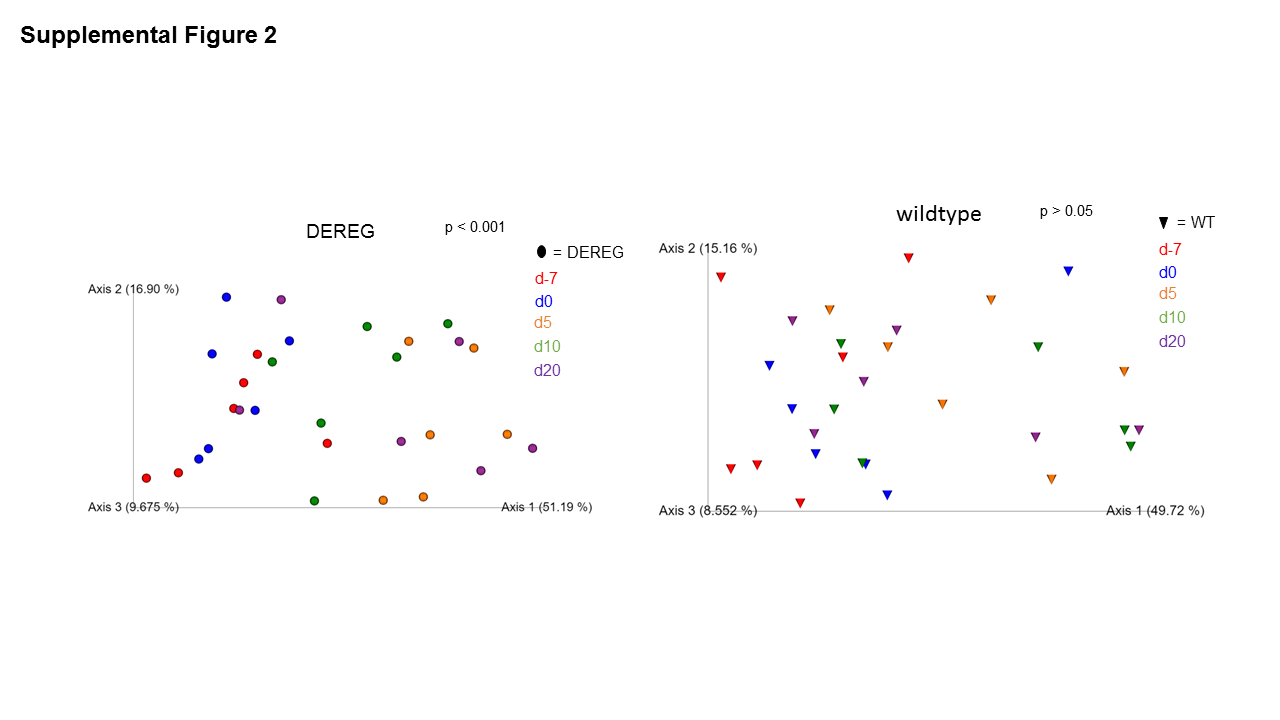

Supplement: Supplementary file 2 — Figure S2. Principal coordinates analysis separates gut microbiota samples of late time points after diphtheria toxin application from time‐points before diphtheria toxin application in DEREG mice but not in wild‐type littermates. [file IMM-159-344-s002.TIF]

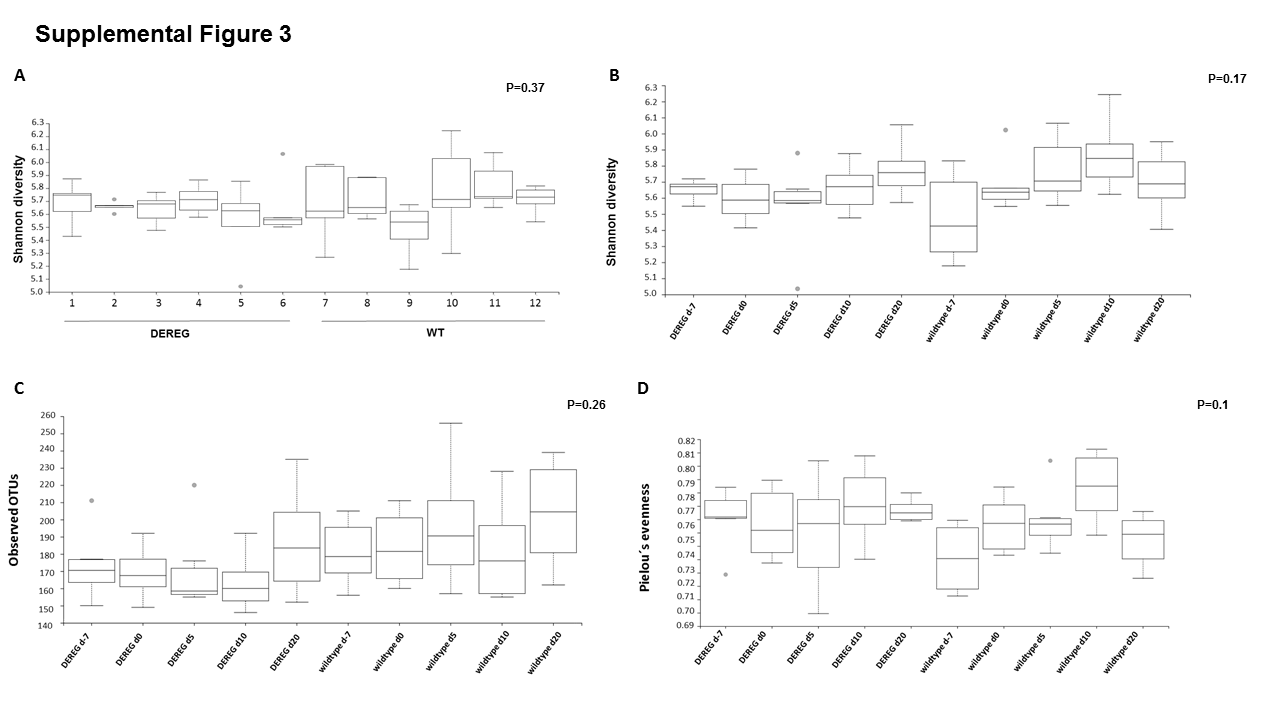

Supplement: Supplementary file 3 — Figure S3. Illustration of α diversity metrics over time in DEREG and wild‐type mice. [file IMM-159-344-s003.TIF]

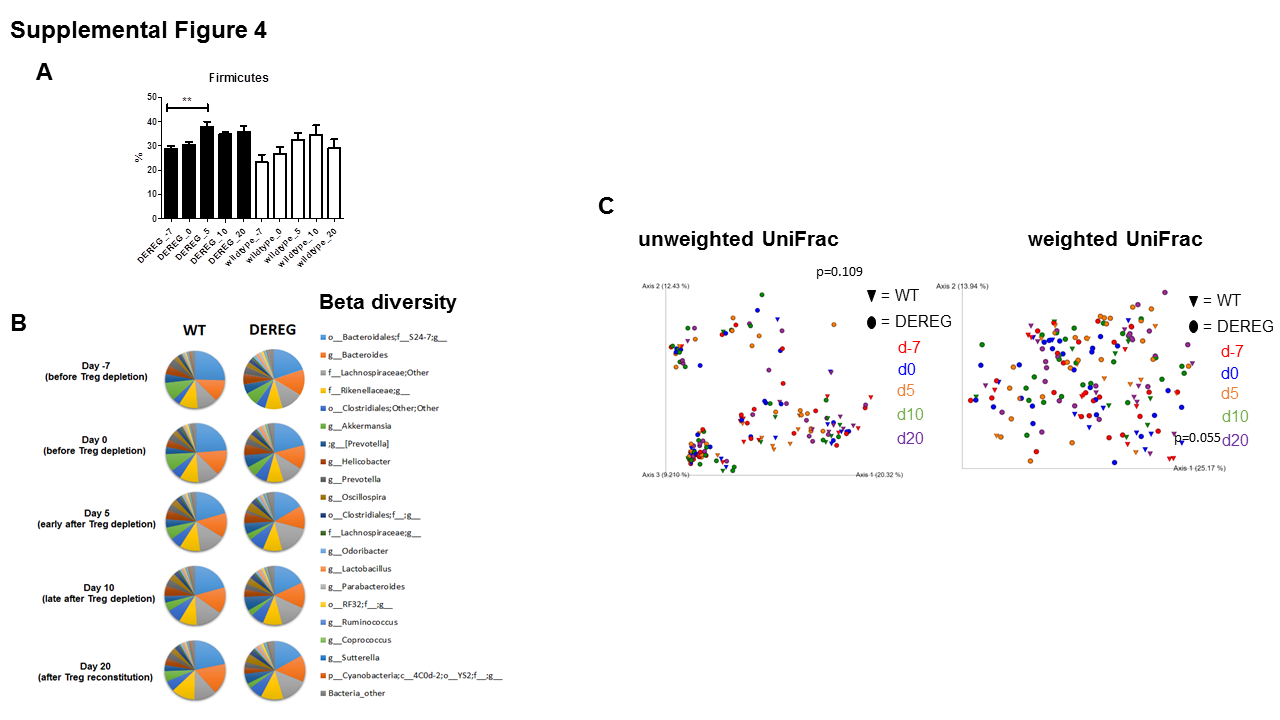

Supplement: Supplementary file 4 — Figure S4. Analysis of the gut microbiota composition and differences in β diversity between 25 DEREG and 11 wild‐type mice of four individual experiments. [file IMM-159-344-s004.TIF]

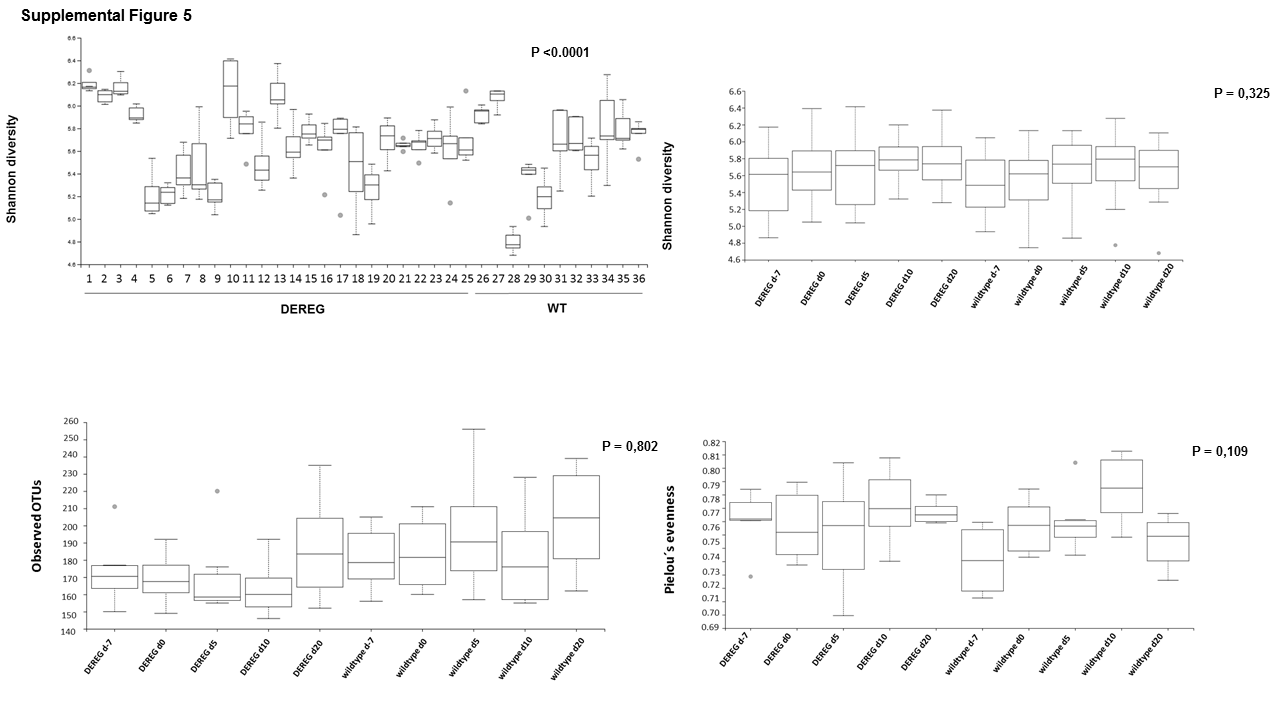

Supplement: Supplementary file 5 — Figure S5. Illustration of α diversity metrics over time in DEREG and wild‐type mice of four individual experiments. [file IMM-159-344-s005.TIF]

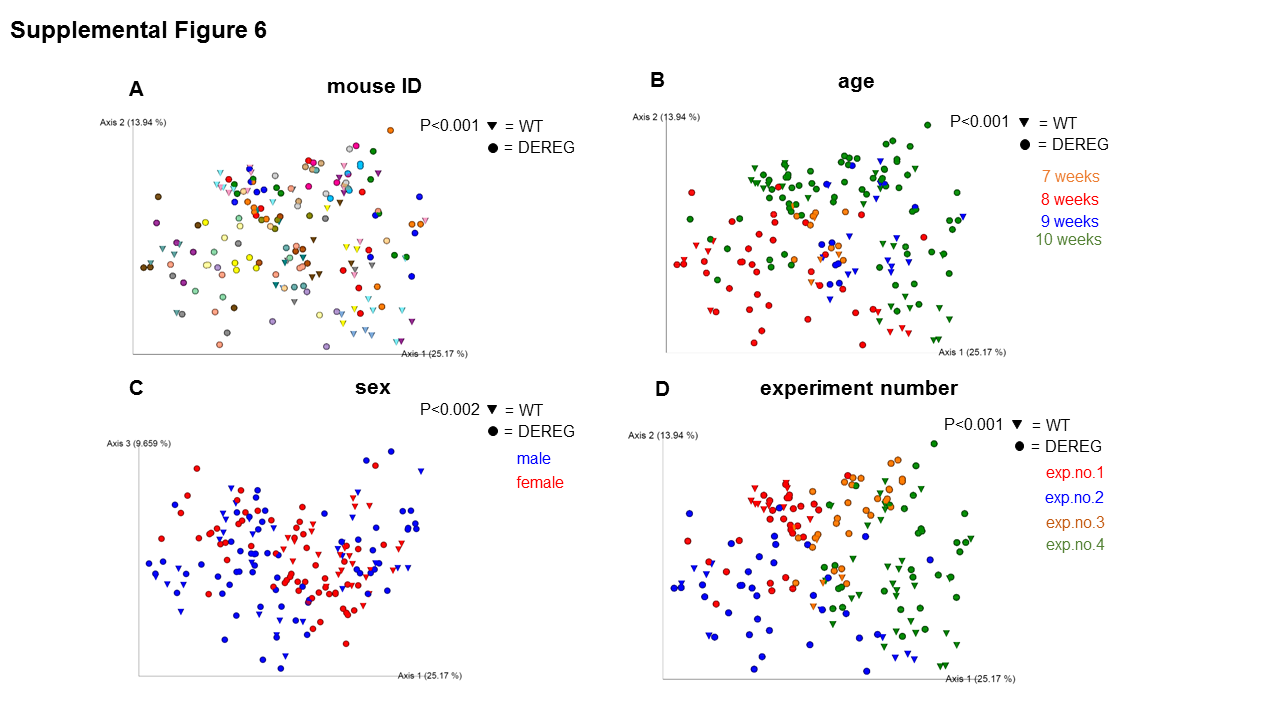

Supplement: Supplementary file 6 — Figure S6. Principal coordinates analysis clusters gut microbiome samples according to individual mouse identifier (ID), age, sex and experiment number. [file IMM-159-344-s006.TIF]

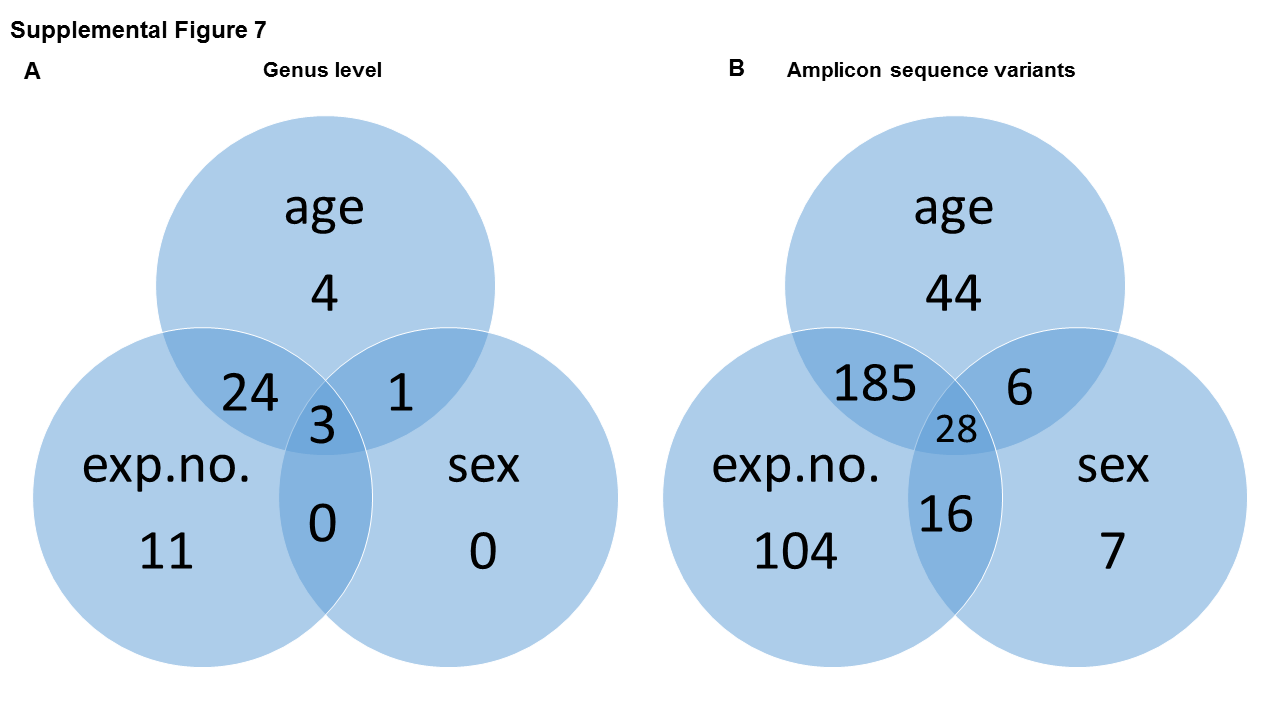

Supplement: Supplementary file 7 — Figure S7. Differentially abundant amplicon sequence variants and genera linked to age, sex and experiment number. [file IMM-159-344-s007.TIF]
