# Supplementary material for: Depletion of Foxp3+ regulatory T cells is accompanied by an increase in the relative abundance of Firmicutes in the murine gut microbiome
Source: Immunology. 2019 Dec 12;159(3):344–53. doi: 10.1111/imm.13158 (PMC7011623; doi:10.1111/imm.13158)
Supplement: Supplementary file 9 — Table S2. Differentially abundant amplicon sequence variants (ASV) according to the variables breeding, sex and cage. [file IMM-159-344-s009.docx]

| **Taxonomy Amplicon Sequence Variant** | **cage** | **breeding** | **sex** |
| --- | --- | --- | --- |
| k__Bacteria | True | True | True |
| k__Bacteria; p__Firmicutes; c__Clostridia; o__Clostridiales; f__; g__; s__ | True | True | True |
| k__Bacteria; p__Bacteroidetes; c__Bacteroidia; o__Bacteroidales; f__Bacteroidaceae; g__Bacteroides | True | True | True |
| k__Bacteria; p__Bacteroidetes; c__Bacteroidia; o__Bacteroidales; f__Rikenellaceae; g__; s__ | True | True | True |
| k__Bacteria; p__Firmicutes; c__Clostridia; o__Clostridiales | True | True | True |
| k__Bacteria; p__Actinobacteria; c__Coriobacteriia; o__Coriobacteriales; f__Coriobacteriaceae; g__Adlercreutzia; s__ | True | True | True |
| k__Bacteria | True | True | True |
| k__Bacteria; p__Cyanobacteria; c__4C0d-2; o__YS2; f__; g__; s__ | True | True | True |
| k__Bacteria; p__Bacteroidetes; c__Bacteroidia; o__Bacteroidales; f__S24-7; g__; s__ | True | True | True |
| k__Bacteria | True | True | True |
| k__Bacteria; p__Firmicutes; c__Clostridia; o__Clostridiales; f__Ruminococcaceae; g__Oscillospira; s__ | True | True | True |
| k__Bacteria; p__Firmicutes; c__Clostridia; o__Clostridiales; f__; g__; s__ | True | True | True |
| k__Bacteria; p__Bacteroidetes; c__Bacteroidia; o__Bacteroidales; f__S24-7; g__; s__ | True | True | True |
| k__Bacteria; p__Actinobacteria; c__Coriobacteriia; o__Coriobacteriales; f__Coriobacteriaceae; g__Adlercreutzia; s__ | True | True | True |
| k__Bacteria; p__Firmicutes; c__Erysipelotrichi; o__Erysipelotrichales; f__Erysipelotrichaceae; g__Coprobacillus; s__ | True | True | True |
| k__Bacteria; p__Bacteroidetes; c__Bacteroidia; o__Bacteroidales; f__Rikenellaceae; g__; s__ | True | True | True |
| k__Bacteria; p__Bacteroidetes; c__Bacteroidia; o__Bacteroidales; f__S24-7; g__; s__ | True | True | True |
| k__Bacteria; p__Firmicutes; c__Clostridia; o__Clostridiales; f__Ruminococcaceae; g__Oscillospira | True | True | True |
| k__Bacteria; p__Bacteroidetes; c__Bacteroidia; o__Bacteroidales; f__Bacteroidaceae; g__Bacteroides; s__ | True | True | False |
| k__Bacteria; p__Bacteroidetes; c__Bacteroidia; o__Bacteroidales; f__Prevotellaceae; g__Prevotella; s__ | True | True | False |
| k__Bacteria; p__Firmicutes; c__Clostridia; o__Clostridiales; f__Lachnospiraceae | True | True | False |
| k__Bacteria | True | True | False |
| k__Bacteria; p__Firmicutes; c__Clostridia; o__Clostridiales; f__Lachnospiraceae | True | True | False |
| k__Bacteria | True | True | False |
| k__Bacteria; p__OD1; c__; o__; f__; g__; s__ | True | True | False |
| k__Bacteria; p__Firmicutes; c__Bacilli; o__Lactobacillales; f__Lactobacillaceae; g__Lactobacillus; s__ | True | True | False |
| k__Bacteria; p__Firmicutes; c__Clostridia; o__Clostridiales; f__; g__; s__ | True | True | False |
| k__Bacteria | True | True | False |
| k__Bacteria; p__Firmicutes; c__Clostridia; o__Clostridiales; f__; g__; s__ | True | True | False |
| k__Bacteria | True | True | False |
| k__Bacteria; p__Firmicutes; c__Clostridia; o__Clostridiales; f__; g__; s__ | True | True | False |
| k__Bacteria | True | True | False |
| k__Bacteria; p__Firmicutes; c__Clostridia; o__Clostridiales; f__Lachnospiraceae; g__; s__ | True | True | False |
| k__Bacteria; p__Firmicutes; c__Clostridia; o__Clostridiales; f__Ruminococcaceae; g__Oscillospira; s__ | True | True | False |
| k__Bacteria; p__Bacteroidetes; c__Bacteroidia; o__Bacteroidales; f__S24-7; g__; s__ | True | True | False |
| k__Bacteria; p__Bacteroidetes; c__Bacteroidia; o__Bacteroidales; f__Rikenellaceae; g__; s__ | True | True | False |
| k__Bacteria; p__Bacteroidetes; c__Bacteroidia; o__Bacteroidales; f__Rikenellaceae; g__; s__ | True | True | False |
| k__Bacteria; p__Bacteroidetes; c__Bacteroidia; o__Bacteroidales; f__Rikenellaceae; g__; s__ | True | True | False |
| k__Bacteria; p__OD1; c__; o__; f__; g__; s__ | True | True | False |
| k__Bacteria; p__Bacteroidetes; c__Bacteroidia; o__Bacteroidales; f__Rikenellaceae; g__; s__ | True | True | False |
| k__Bacteria; p__Bacteroidetes; c__Bacteroidia; o__Bacteroidales | True | True | False |
| k__Bacteria; p__Firmicutes; c__Clostridia; o__Clostridiales | True | True | False |
| k__Bacteria; p__Firmicutes; c__Clostridia; o__Clostridiales; f__; g__; s__ | True | True | False |
| k__Bacteria; p__Firmicutes; c__Clostridia; o__Clostridiales; f__Ruminococcaceae; g__Anaerotruncus; s__ | True | True | False |
| k__Bacteria; p__Firmicutes; c__Erysipelotrichi; o__Erysipelotrichales; f__Erysipelotrichaceae; g__; s__ | True | True | False |
| k__Bacteria; p__Firmicutes; c__Clostridia; o__Clostridiales; f__Lachnospiraceae | True | True | False |
| k__Bacteria; p__Firmicutes; c__Clostridia; o__Clostridiales; f__Ruminococcaceae; g__Oscillospira; s__ | True | True | False |
| k__Bacteria; p__Firmicutes; c__Clostridia; o__Clostridiales; f__; g__; s__ | True | True | False |
| k__Bacteria; p__Firmicutes; c__Clostridia; o__Clostridiales; f__; g__; s__ | True | True | False |
| k__Bacteria; p__Firmicutes; c__Clostridia; o__Clostridiales | True | True | False |
| k__Bacteria; p__Verrucomicrobia; c__Verrucomicrobiae; o__Verrucomicrobiales; f__Verrucomicrobiaceae; g__Akkermansia; s__muciniphila | True | True | False |
| k__Bacteria; p__Firmicutes; c__Clostridia; o__Clostridiales | True | True | False |
| k__Bacteria; p__Verrucomicrobia; c__Verrucomicrobiae; o__Verrucomicrobiales; f__Verrucomicrobiaceae; g__Akkermansia; s__muciniphila | True | True | False |
| k__Bacteria; p__Verrucomicrobia; c__Verrucomicrobiae; o__Verrucomicrobiales; f__Verrucomicrobiaceae; g__Akkermansia; s__muciniphila | True | True | False |
| k__Bacteria; p__Bacteroidetes; c__Bacteroidia; o__Bacteroidales; f__S24-7; g__; s__ | True | True | False |
| k__Bacteria; p__Firmicutes; c__Clostridia; o__Clostridiales | True | True | False |
| k__Bacteria; p__Firmicutes; c__Clostridia; o__Clostridiales | True | True | False |
| k__Bacteria; p__Firmicutes; c__Clostridia; o__Clostridiales; f__; g__; s__ | True | True | False |
| k__Bacteria; p__Firmicutes; c__Clostridia; o__Clostridiales | True | True | False |
| k__Bacteria; p__Firmicutes; c__Clostridia; o__Clostridiales | True | True | False |
| k__Bacteria; p__Tenericutes; c__Mollicutes; o__RF39; f__; g__; s__ | True | True | False |
| k__Bacteria; p__OD1; c__; o__; f__; g__; s__ | True | True | False |
| k__Bacteria; p__Bacteroidetes; c__Bacteroidia; o__Bacteroidales; f__Bacteroidaceae; g__Bacteroides; s__ | True | True | False |
| k__Bacteria; p__Firmicutes; c__Clostridia; o__Clostridiales; f__Lachnospiraceae | True | True | False |
| k__Bacteria; p__Bacteroidetes; c__Bacteroidia; o__Bacteroidales; f__S24-7; g__; s__ | True | True | False |
| k__Bacteria; p__Firmicutes; c__Clostridia; o__Clostridiales | True | True | False |
| k__Bacteria; p__Bacteroidetes; c__Bacteroidia; o__Bacteroidales; f__S24-7; g__; s__ | True | True | False |
| k__Bacteria; p__Proteobacteria; c__Alphaproteobacteria; o__Rickettsiales; f__mitochondria | True | True | False |
| k__Bacteria; p__Bacteroidetes; c__Bacteroidia; o__Bacteroidales; f__Bacteroidaceae; g__Bacteroides; s__ | True | True | False |
| k__Bacteria; p__Firmicutes; c__Clostridia; o__Clostridiales; f__; g__; s__ | True | True | False |
| k__Bacteria | True | True | False |
| k__Bacteria; p__Firmicutes; c__Clostridia; o__Clostridiales | True | True | False |
| k__Bacteria; p__Bacteroidetes; c__Bacteroidia; o__Bacteroidales; f__Bacteroidaceae; g__Bacteroides; s__ | True | True | False |
| k__Bacteria; p__Bacteroidetes; c__Bacteroidia; o__Bacteroidales; f__S24-7; g__; s__ | True | True | False |
| k__Bacteria; p__OD1; c__; o__; f__; g__; s__ | True | True | False |
| k__Bacteria; p__Bacteroidetes; c__Bacteroidia; o__Bacteroidales; f__S24-7; g__; s__ | True | True | False |
| k__Bacteria | True | True | False |
| k__Bacteria; p__Firmicutes; c__Clostridia; o__Clostridiales; f__; g__; s__ | True | True | False |
| k__Bacteria; p__Cyanobacteria; c__4C0d-2; o__YS2; f__; g__; s__ | True | True | False |
| k__Bacteria; p__Bacteroidetes; c__Bacteroidia; o__Bacteroidales; f__Rikenellaceae | True | True | False |
| k__Bacteria; p__Bacteroidetes; c__Bacteroidia; o__Bacteroidales; f__Rikenellaceae | True | True | False |
| k__Bacteria; p__Firmicutes; c__Clostridia; o__Clostridiales; f__Lachnospiraceae | True | True | False |
| k__Bacteria; p__Bacteroidetes; c__Bacteroidia; o__Bacteroidales; f__S24-7; g__; s__ | True | True | False |
| k__Bacteria; p__Firmicutes; c__Clostridia; o__Clostridiales; f__Lachnospiraceae | True | True | False |
| k__Bacteria; p__Bacteroidetes; c__Bacteroidia; o__Bacteroidales; f__S24-7; g__; s__ | True | True | False |
| k__Bacteria | True | True | False |
| k__Bacteria; p__Firmicutes; c__Clostridia; o__Clostridiales; f__Lachnospiraceae; g__; s__ | True | True | False |
| k__Bacteria; p__Firmicutes; c__Clostridia; o__Clostridiales; f__; g__; s__ | True | True | False |
| k__Bacteria; p__Bacteroidetes; c__Bacteroidia; o__Bacteroidales; f__Bacteroidaceae; g__Bacteroides; s__acidifaciens | True | True | False |
| k__Bacteria; p__Bacteroidetes; c__Bacteroidia; o__Bacteroidales; f__Rikenellaceae; g__; s__ | True | True | False |
| k__Bacteria | True | True | False |
| k__Bacteria; p__Firmicutes; c__Clostridia; o__Clostridiales | True | True | False |
| k__Bacteria; p__Proteobacteria; c__Gammaproteobacteria; o__Pseudomonadales; f__Pseudomonadaceae; g__Pseudomonas | True | True | False |
| k__Bacteria | True | True | False |
| k__Bacteria; p__Bacteroidetes; c__Bacteroidia; o__Bacteroidales; f__Bacteroidaceae; g__Bacteroides; s__ | True | True | False |
| k__Bacteria; p__Firmicutes; c__Clostridia; o__Clostridiales | True | True | False |
| k__Bacteria | True | True | False |
| k__Bacteria | True | True | False |
| k__Bacteria; p__Firmicutes; c__Clostridia; o__Clostridiales | True | True | False |
| k__Bacteria; p__Cyanobacteria; c__4C0d-2; o__YS2; f__; g__; s__ | True | True | False |
| k__Bacteria; p__Bacteroidetes; c__Bacteroidia; o__Bacteroidales | True | True | False |
| k__Bacteria; p__Bacteroidetes; c__Bacteroidia; o__Bacteroidales; f__Rikenellaceae; g__; s__ | True | True | False |
| k__Bacteria; p__Proteobacteria; c__Betaproteobacteria; o__Burkholderiales; f__Alcaligenaceae; g__Sutterella; s__ | True | True | False |
| k__Bacteria; p__Bacteroidetes; c__Bacteroidia; o__Bacteroidales; f__Porphyromonadaceae; g__Parabacteroides; s__distasonis | True | True | False |
| k__Bacteria; p__Bacteroidetes; c__Bacteroidia; o__Bacteroidales | True | True | False |
| k__Bacteria; p__Firmicutes; c__Clostridia; o__Clostridiales; f__Lachnospiraceae; g__Robinsoniella; s__peoriensis | True | True | False |
| k__Bacteria; p__Firmicutes; c__Clostridia; o__Clostridiales; f__; g__; s__ | True | False | True |
| k__Bacteria; p__Firmicutes; c__Clostridia; o__Clostridiales | True | False | True |
| k__Bacteria; p__Firmicutes; c__Clostridia; o__Clostridiales; f__Ruminococcaceae | True | False | True |
| k__Bacteria; p__Proteobacteria; c__Alphaproteobacteria; o__Rickettsiales; f__mitochondria | True | False | True |
| k__Bacteria; p__Firmicutes; c__Clostridia; o__Clostridiales | True | False | True |
| k__Bacteria; p__Bacteroidetes; c__Bacteroidia; o__Bacteroidales; f__Rikenellaceae; g__Alistipes | True | False | True |
| k__Bacteria; p__Bacteroidetes; c__Bacteroidia; o__Bacteroidales; f__Bacteroidaceae; g__Bacteroides; s__ | True | False | True |
| k__Bacteria; p__OD1; c__; o__; f__; g__; s__ | True | False | False |
| k__Bacteria; p__Bacteroidetes; c__Bacteroidia; o__Bacteroidales; f__S24-7; g__; s__ | True | False | False |
| k__Bacteria; p__Bacteroidetes; c__Bacteroidia; o__Bacteroidales; f__S24-7; g__; s__ | True | False | False |
| k__Bacteria; p__Bacteroidetes; c__Bacteroidia; o__Bacteroidales; f__Bacteroidaceae; g__Bacteroides; s__acidifaciens | True | False | False |
| k__Bacteria; p__Firmicutes; c__Clostridia; o__Clostridiales; f__; g__; s__ | True | False | False |
| k__Bacteria; p__Bacteroidetes; c__Bacteroidia; o__Bacteroidales; f__Rikenellaceae; g__; s__ | True | False | False |
| k__Bacteria; p__Firmicutes; c__Bacilli; o__Lactobacillales; f__Lactobacillaceae; g__Lactobacillus; s__ | True | False | False |
| k__Bacteria; p__Firmicutes; c__Clostridia; o__Clostridiales; f__Ruminococcaceae; g__Anaerotruncus; s__ | True | False | False |
| k__Bacteria; p__Firmicutes; c__Clostridia; o__Clostridiales; f__Lachnospiraceae | True | False | False |
| k__Bacteria; p__Firmicutes; c__Clostridia; o__Clostridiales | True | False | False |
| k__Bacteria; p__Firmicutes; c__Clostridia; o__Clostridiales | True | False | False |
| k__Bacteria; p__Firmicutes; c__Clostridia; o__Clostridiales | True | False | False |
| k__Bacteria; p__Proteobacteria; c__Epsilonproteobacteria; o__Campylobacterales; f__Helicobacteraceae; g__Helicobacter | True | False | False |
| k__Bacteria; p__Firmicutes; c__Clostridia; o__Clostridiales; f__Lachnospiraceae | True | False | False |
| k__Bacteria; p__Firmicutes; c__Clostridia; o__Clostridiales | True | False | False |
| k__Bacteria; p__Bacteroidetes; c__Bacteroidia; o__Bacteroidales; f__Porphyromonadaceae; g__Parabacteroides; s__ | True | False | False |
| k__Bacteria; p__Firmicutes; c__Clostridia; o__Clostridiales; f__Lachnospiraceae | True | False | False |
| k__Bacteria; p__Firmicutes; c__Clostridia; o__Clostridiales; f__Lachnospiraceae | True | False | False |
| k__Bacteria; p__Firmicutes; c__Clostridia; o__Clostridiales; f__; g__; s__ | True | False | False |
| k__Bacteria; p__Firmicutes; c__Clostridia; o__Clostridiales; f__Lachnospiraceae; g__Coprococcus; s__ | True | False | False |
| k__Bacteria | True | False | False |
| k__Bacteria; p__Bacteroidetes; c__Bacteroidia; o__Bacteroidales; f__S24-7; g__; s__ | True | False | False |
| k__Bacteria; p__Verrucomicrobia; c__Verrucomicrobiae; o__Verrucomicrobiales; f__Verrucomicrobiaceae; g__Akkermansia; s__muciniphila | True | False | False |
| k__Bacteria; p__Bacteroidetes; c__Bacteroidia; o__Bacteroidales; f__S24-7; g__; s__ | True | False | False |
| k__Bacteria; p__Firmicutes; c__Clostridia; o__Clostridiales; f__Lachnospiraceae | True | False | False |
| k__Bacteria; p__Bacteroidetes; c__Bacteroidia; o__Bacteroidales; f__Bacteroidaceae; g__Bacteroides | True | False | False |
| k__Bacteria | True | False | False |
| k__Bacteria | True | False | False |
| k__Bacteria | True | False | False |
| k__Bacteria; p__Firmicutes; c__Clostridia; o__Clostridiales; f__; g__; s__ | True | False | False |
| k__Bacteria; p__Firmicutes; c__Clostridia; o__Clostridiales | True | False | False |
| k__Bacteria; p__Bacteroidetes; c__Bacteroidia; o__Bacteroidales; f__[Paraprevotellaceae]; g__[Prevotella]; s__ | True | False | False |
| k__Bacteria; p__Bacteroidetes; c__Bacteroidia; o__Bacteroidales; f__S24-7; g__; s__ | True | False | False |
| k__Bacteria; p__Bacteroidetes; c__Bacteroidia; o__Bacteroidales; f__Bacteroidaceae; g__Bacteroides; s__ | True | False | False |
| k__Bacteria; p__Proteobacteria; c__Alphaproteobacteria; o__Rickettsiales; f__mitochondria | True | False | False |
| k__Bacteria; p__Firmicutes; c__Clostridia; o__Clostridiales; f__Lachnospiraceae | True | False | False |
| k__Bacteria | True | False | False |
| k__Bacteria | True | False | False |
| k__Bacteria | True | False | False |
| k__Bacteria; p__Firmicutes; c__Bacilli; o__Lactobacillales; f__Lactobacillaceae; g__Lactobacillus; s__reuteri | True | False | False |
| k__Bacteria | True | False | False |
| k__Bacteria; p__Firmicutes; c__Clostridia; o__Clostridiales; f__Lachnospiraceae; g__; s__ | True | False | False |
| k__Bacteria; p__Firmicutes; c__Clostridia; o__Clostridiales; f__Ruminococcaceae; g__Oscillospira; s__ | True | False | False |
| k__Bacteria | True | False | False |
| k__Bacteria; p__Proteobacteria; c__Deltaproteobacteria; o__Desulfovibrionales; f__Desulfovibrionaceae; g__Desulfovibrio; s__C21_c20 | True | False | False |
| k__Bacteria | True | False | False |
| k__Bacteria; p__Bacteroidetes; c__Bacteroidia; o__Bacteroidales; f__Bacteroidaceae; g__Bacteroides; s__ | True | False | False |
| k__Bacteria; p__Firmicutes; c__Clostridia; o__Clostridiales | True | False | False |
| k__Bacteria; p__Firmicutes; c__Bacilli; o__Lactobacillales; f__Lactobacillaceae; g__Lactobacillus; s__ | True | False | False |
| k__Bacteria; p__Firmicutes; c__Clostridia; o__Clostridiales; f__; g__; s__ | True | False | False |
| k__Bacteria; p__Bacteroidetes; c__Bacteroidia; o__Bacteroidales; f__S24-7; g__; s__ | True | False | False |
| k__Bacteria; p__Bacteroidetes; c__Bacteroidia; o__Bacteroidales; f__S24-7; g__; s__ | True | False | False |
| k__Bacteria; p__Actinobacteria; c__Coriobacteriia; o__Coriobacteriales; f__Coriobacteriaceae; g__Adlercreutzia; s__ | True | False | False |
| k__Bacteria; p__Firmicutes; c__Clostridia; o__Clostridiales; f__; g__; s__ | True | False | False |
| k__Bacteria; p__Firmicutes; c__Clostridia; o__Clostridiales; f__; g__; s__ | True | False | False |
| k__Bacteria; p__Bacteroidetes; c__Bacteroidia; o__Bacteroidales; f__S24-7; g__; s__ | False | True | False |
| k__Bacteria; p__Firmicutes; c__Clostridia; o__Clostridiales; f__; g__; s__ | False | True | False |
| k__Bacteria; p__Cyanobacteria; c__4C0d-2; o__YS2; f__; g__; s__ | False | True | False |
| k__Bacteria; p__Cyanobacteria; c__4C0d-2; o__YS2; f__; g__; s__ | False | True | False |
| k__Bacteria; p__Firmicutes; c__Clostridia; o__Clostridiales | False | True | False |
| k__Bacteria; p__Firmicutes; c__Clostridia; o__Clostridiales | False | True | False |
| k__Bacteria; p__Bacteroidetes; c__Bacteroidia; o__Bacteroidales; f__S24-7; g__; s__ | False | True | False |
| k__Bacteria; p__Firmicutes; c__Clostridia; o__Clostridiales; f__Lachnospiraceae; g__; s__ | False | True | False |
| k__Bacteria | False | True | False |
| k__Bacteria; p__Cyanobacteria; c__Chloroplast; o__Streptophyta; f__; g__; s__ | False | True | False |
| k__Bacteria; p__Bacteroidetes; c__Bacteroidia; o__Bacteroidales; f__S24-7; g__; s__ | False | True | False |
| k__Bacteria; p__Bacteroidetes; c__Bacteroidia; o__Bacteroidales; f__S24-7; g__; s__ | False | True | False |
